# Antiphospholipid Antibodies and Systemic Scleroderma

**DOI:** 10.4274/tjh.2012.0059

**Published:** 2013-03-05

**Authors:** Awa Oumar Touré, Fatimata Ly, Abibatou Sall, Alassane Diatta, Macoura Gadji, Moussa Seck, Blaise Faye, Tandakha Dieye, Saliou Diop

**Affiliations:** 1 laboratoire d’hématologie; Université Cheikh Anta Diop de Dakar (UCAD); 2 Service de dermatologie UCAD; 3 Laboratoire de biochimie UCAD

**Keywords:** Antiphospholipids, Systemic scleroderma, complication, Senegal

## Abstract

**Objective:** Antiphospholipid antibodies (APLs) could be associated with an increased risk of vascular pathologies in systemic scleroderma. The aim of our study was to search for APLs in patients affected by systemic scleroderma and to evaluate their involvement in the clinical manifestations of this disease.

**Materials and Methods:** We conducted a cross-sectional descriptive study, from January 2009 until August 2010, with patients received at the Department of Dermatology (Dakar, Senegal). Blood samples were taken at the hematology laboratory and were analyzed for the presence of APLs.

**Results:** Forty patients were recruited. Various types of either isolated or associated APLs were found in 23 patients, i.e. 57.5% of the study population. The most frequently encountered antibody was IgG anti-β2 GPI (37.5% of the patients), followed by anticardiolipins (17.5%) and lupus anticoagulants (5%). No statistically significant association of positive antiphospholipid-related tests to any of the scleroderma complications could be demonstrated.

**Conclusion:** A high proportion of patients showing association of systemic scleroderma and APLs suggests the presence of a morbid correlation between these 2 pathologies. It would be useful to follow a cohort of patients affected by systemic scleroderma in order to monitor vascular complications following confirmation of the presence of antiphospholipid syndrome.

**Conflict of interest:**None declared.

## Abstract (tr)

**Amaç:** Antifosfolipid antikorlar (AA) sistemik sklerodermada görülen damarsal patolojiler açısından artmış risk oluşturabilir. Çalışmamızın amacı sistemik skleroderma hastalarında AA tayini yapılması ve hastalığın klinik özellikleri üzerinde bu antikorların etkisinin değerlendirilmesidir.

**Yöntemler:** Ocak 2009 ile Ağustos 2010 tarihleri arasında Dermatoloji Kliniğine (Dakar, Senagal) başvuran hastalar arasında kesitsel betimleyici bir çalışma planladık. Hastaların kan örnekleri hematoloji laboratuvarında alındı ve AA varlığı açısından incelendi.

**Bulgular:** Kırk hasta çalışmaya dahil edildi. Çalışma popülasyonundan 23 hastada (%57,5) izole ve birden fazla AA tespit edildi. En sık rastlanan antikor IgG anti-β2 GPI idi (%37,5), bunu sırasıyla antikardiyolipinler (%17,5) ve lupus antikoagülanı (%5) izlemekteydi. Skleroderma ile ilgili herhangi bir komplikasyon ile pozitif antifosfolipid testler arasında pozitif anlamlı bir ilişki gösterilemedi.

**Sonuç:** Skleroderma ve AA birlikteliğinin yüksek oranda bulunması, bu iki patoloji arasında morbid bir korelasyon varlığını düşündürmektedir. Sistemik skleroderma tanısı olan hastaların antifosfolipid sendrom varlığı tespit edilmesi sonrasında damarsal komplikasyonlar açısından takip edilmeleri yararlı olacaktır. Bulgular: Kırk hasta çalışmaya dahil edildi. Çalışma popülasyonundan 23 hastada (%57,5) izole ve birden fazla AA tespit edildi. En sık rastlanan antikor IgG anti-β2 GPI idi (%37,5), bunu sırasıyla antikardiyolipinler (%17,5) ve lupus antikoagülanı (%5) izlemekteydi. Skleroderma ile ilgili herhangi bir komplikasyon ile pozitif antifosfolipid testler arasında pozitif anlamlı bir ilişki gösterilemedi. Sonuç: Skleroderma ve AA birlikteliğinin yüksek oranda bulunması, bu iki patoloji arasında morbid bir korelasyon varlığını düşündürmektedir. Sistemik skleroderma tanısı olan hastaların antifosfolipid sendrom varlığı tespit edilmesi sonrasında damarsal komplikasyonlar açısından takip edilmeleri yararlı olacaktır.

## INTRODUCTION

Antiphospholipid antibodies (APLs) are a family of antibodies against phospholipids, against the proteins that bind the phospholipids (also referred to as cofactors), or against both of these; they can have a pathogenic effect by interfering with the membrane phospholipids of the endothelial cells and the platelets, or with the phospholipids involved in the coagulation cascade. The most clinically important APLs are the lupus anticoagulant, the anticardiolipin antibodies, and anti-β2 GPI.

These antibodies are either isolated or, most commonly, are associated with an autoimmune disease, in particular systemic lupus, systemic scleroderma (SS), and antiphospholipid syndrome.

SS is an autoimmune disease with vascular involvement characterized by microangiopathy, endothelial injury, and diffuse sclerosis of unexplained mechanism. The vascular component is best exemplified by Raynaud’s phenomenon, the most frequent manifestation of SS.

The APLs are thought to be associated with an increased risk of vascular pathologies in SS [1].

The aim of our study was to look for APLs in patients affected by SS and to evaluate their involvement in the clinical manifestations of this systemic disease 

## MATERIALS AND METHODS

We conducted a cross-sectional descriptive study from January 2009 until August 2010.

The patients were recruited from the Department of Dermatology and the analyses were carried out at the hematology laboratory of the Aristide Le Dantec Hospital.

Inclusion criteria were as follows:

• Patients presenting with typical skin manifestations (tightness, thickening, and/or non-pitting induration of the skin affecting the face, neck, torso, and proximal portions of the upper and lower extremities) or with the presence of at least 2 of the following: sclerodactyly, stellate pulp scars or ulceration of the fingertips, or bilateral pulmonary fibrosis, mainly at the lung bases.

• Patients with mixed connective tissue disease associating SS with dermatomyositis, Sharp syndrome, or Gougerot-Sjögren syndrome.

• Patient’s willingness to participate in the study.

Once the diagnosis had been made by the dermatologist, the patient was sent to the laboratory for the required samples to be taken.

Physical examination, blood analyses, and imaging as required were done to screen the patients for disease-related cutaneous (Raynaud’s phenomenon, cutaneous sclerosis, hypochromic spots, skin calcinosis, stellate scars, ulcerations, chronic ulcers), cardiovascular (loud S2), pleuropulmonary (interstitial lung disease, chronic fibrosis, pulmonary arterial hypertension), and gastrointestinal (dysphagia, signs of intestinal malabsorption) signs and symptoms. 

The following biological parameters were analyzed:

• Activated partial thromboplastin time (aPTT) was examined using the chronometric technique with a compact STA analyzer from Diagnostica Stago Laboratories (France). The aPTT was considered to be prolonged when the ratio of the patient’s time to control time was greater than 1.2. We then performed an aPTT mixing test and selected all samples without correction for lupus anticoagulant screening. 

• The anticardiolipin and the anti-β2 glycoprotein IgG/IgM antibody levels were measured by ELISA using the ASSERACHROM APA IgM and IgG and ASSERACHROM anti-β2GP IgG and IgM reagents from Diagnostica Stago Laboratories. All values above 40 UPL/mL were accepted as positive.

• The lupus anticoagulant (screening and confirmation) was tested via the chronometric method on the compact STA analyzer using the following reagents from Diagnostica Stago Laboratories: STA STACLOT DRVV screen and STA STACLOT DRVV confirm. 

The clotting time (CT) determination of the plasma samples was carried out immediately after blood sampling. The final result was expressed as: Screen ratio = screen CT of the plasma to be tested / screen CT of the reference pool.

The result was accepted as abnormal if the screen ratio was higher than 1.2 and the presence of lupus anticoagulant was suspected. In suspected cases, a second CT determination (confirmatory CT) was done. Confirmation ratio was calculated (=confirm CT plasma to be tested / confirm CT of reference pool). Thereafter, the normalized ratio was computed using the following formula: Normalized ratio = screen ratio / confirm ratio. A normalized ratio of equal to or higher than 1.2 confirmed the presence of lupus anticoagulant.

Recording and statistical analysis of the data was done using version 3.5.1 (2008) of the Epi Info software. Fischer’s test was used to compare the data. P values of less than 0.05 were accepted as statistically significant.

## RESULTS

**Overall Results**

**• Epidemiological and Clinical Findings**

Forty patients were recruited during the study period out of a total of 19,340 hospitalized patients, thus corresponding to a hospital prevalence of 2.07‰ for SS.

The median age of these patients was 41 years, with a range of 18 and 68 years and a male-to-female sex ratio of 0.38.

The most common presenting symptom was hypochromic skin macula (100% of the patients), which was associated with cutaneous sclerosis in 92% of the cases. Patients were usually diagnosed at least 1 year after emergence of the symptoms.

Some patients also presented with vascular events such as venous or arterial thrombosis (cutaneous thrombosis, pulmonary embolism) and hypertension. 

Physical examination and imaging studies revealed Raynaud’s phenomenon, stellate scars, sclerodactyly, pulmonary fibrosis, pulmonary hypertension, and cardiac arrhythmias in a majority of patients (Table 1).

**• Laboratory Findings**

Seven patients (17.5%) had a prolonged aPTT, which was not corrected by the addition of normal plasma.

Various types of isolated or associated APLs were found and confirmed at least 12 weeks later in 23 patients, i.e. 57.5% of the study population. 

The most frequently encountered antibody was anti-β2 GPI (50% of the patients), followed by anticardiolipins (17.5%) and lupus anticoagulants (5%).

o Anti-β2 GPI 

Fifteen patients had IgG and 13 had IgM. Eight patients had both isotypes.

o Anticardiolipins

IgG and IgM anticardiolipins were detected in 7 and 2 patients, respectively.

o Lupus anticoagulant 

The presence of lupus anticoagulant was confirmed in 2 patients (Table 2).

Eight patients had at least 2 types of APLs (anticardiolipin or lupus anticoagulant associated with anti-β2 GPI). Two of them developed pulmonary hypertension and 2 others developed cutaneous ulcerations. 

**• Analytical results**

With uncorrected mixing study, 90% of patients were not carriers of APLs.

Three of the 4 patients with a family history of venous thromboembolism had APLs; however, the correlation was insignificant.

The presence of APLs indicated a tendency for some of the SS complications, in particular Raynaud’s phenomenon, cutaneous ulcerations, and pulmonary hypertension. However, the correlation remained insignificant (Table 3).

## DISCUSSION

The APLs are autoantibodies, which are thought to be responsible for a real autoimmune pathology: the primary antiphospholipid syndrome described by Hughes in 1987 [2]. These APLs can also occur in the context of other autoimmune diseases having the secondary antiphospholipid syndrome [2].

Lupus anticoagulant was first defined in patients with systemic lupus erythematosus as an inhibitor of coagulation leading paradoxically to thrombosis.

Since then, immunoenzymology has led, and still leads, to the discovery of new APLs. Those considered to be pathogenic, according to the last consensus conference held in Sydney [3], are the lupus anticoagulant, anti-β2 GPI, and anticardiolipins.

Numerous studies have shown that antiphospholipids are associated with autoimmune diseases [4,5,6,7]. SS, a rare autoimmune disease [8], has vascular complications that are occasionally similar to those encountered with the antiphospholipid syndrome.

Over a period of 8 months, we screened 40 SS patients for the presence of APLs. The patients were mainly young women (mean age: 41 years, sex ratio: 0.38). Unlike in the Caucasian population [8], where the onset of disease occurs at advanced ages, the median age of occurrence in our cohort, being 41 years, was in line with that found in Morocco and in sub-Saharan Africa [9].

The disparity between the significance of different types of antiphospholipids encountered does not allow a reliable comparison to be made among the limited studies performed on this subject.

Overall, we found a 57.5% prevalence of APLs, which is considerably higher than that previously found in Italy by Picillo et al. [10] and in Japan by Ihn et al. [11]. These discrepancies could be explained by the low level of sensitivity and specificity of the techniques used during the 1990s. 

It was only towards the mid-2000s that research, including that of the present authors, specifically began to address the different types of APLs.

Anticardiolipins, mainly IgG, were found in significant quantities in 17.5% of our patients. This frequency, similar to that found in France by Marie et al. [6], is lower than that found by Ihn et al. in Japan [11] and by Picillo et al. in Europe [10].

As for anti-β2 GPIs, their prevalence in our study population was close to that observed in Japan, but greater than that found in the Caucasian population.

The lupus anticoagulant is considered by some authors to be associated with an increased risk of venous or arterial thrombosis [12].Despite limitations associated with our work (low number of patients, lack of comparison with a healthy population), we may say that 57.5% of patients affected by SS had at least one type of APL. The presence of APLs has been reported to be associated with certain complications of scleroderma: glomerular nephropathy [5,7], cutaneous ulcerations [6], pulmonary hypertension [6], deep venous thrombosis [13], and pulmonary fibrosis [1]. As a result of the small number of studied cases, we were not able to find a significant difference between carrier and non-carrier patients. 

As in previous studies, we did not find a statistically significant relationship between the presence of APLs and the occurrence of scleroderma complications. However, the high proportion of patients showing association of SS and APLs suggests the presence of a morbid correlation between these 2 pathologies. It would be interesting to follow a large cohort of patients affected by SS in order to search for vascular complications, following the confirmed presence of antiphospholipid syndrome.

**Conflict of Interest Statement**

The authors of this paper have no conflicts of interest, including specific financial interests, relationships, and/or affiliations relevant to the subject matter or materials included.

## Figures and Tables

**Table 1:**
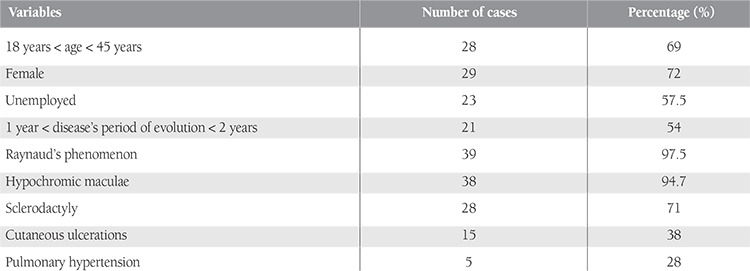
Epidemiological and clinical variables.

**Table 2:**
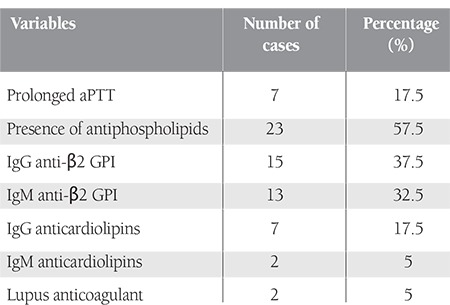
Summary of biological results.

**Table 3:**
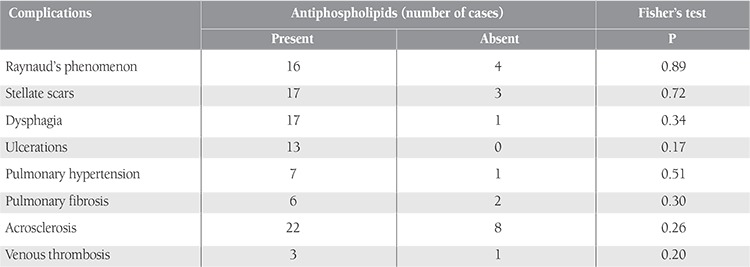
Correlation between antiphospholipids and scleroderma complications.
